# Characteristics of soil organic carbon and its components under long-term different crop rotation patterns

**DOI:** 10.1371/journal.pone.0336581

**Published:** 2025-11-21

**Authors:** Xingzhe Zhang, Chunguang Liu, Baicheng Wang, Dehai Xu, Xinrui Shi, Shuai Zhang, Bing Yang, Wenhui Wang, Xianghai Meng

**Affiliations:** Mudanjiang Branch, Heilongjiang Academy of Agricultural Sciences, Mudanjiang, China; Zhejiang Agriculture and Forestry University: Zhejiang A and F University, CHINA

## Abstract

To clarify the effects of different crop rotation patterns on soil organic carbon content and its components, four treatments were set up: corn continuous cropping, soybean continuous cropping, corn-soybean rotation, and corn-soybean-potato rotation. The relationship between soil organic carbon, active carbon, total nitrogen content, and soil aggregates in the 0–50 cm soil layer was analyzed. The results showed that the corn-soybean-potato rotation treatment significantly improved the stability of soil aggregates, with a water stable aggregate content of>0.25 mm increasing by 23.8% −66.3% compared to continuous cropping. Crop rotation increased the soil structure index of the 0–30 cm soil layer, reaching a maximum of 99.60. Among the activated carbon components, the easily oxidizable carbon content was highest in crop rotation treatment, which increased by 31.8% compared to continuous cropping.The total organic carbon and total nitrogen content in the soil were highest in the corn continuous cropping treatment. The above results indicated that crop rotation can improve soil structure and enhance aggregate stability, but its impact on soil carbon and nitrogen storage is relatively small.

## Introduction

Black soil is an important arable land resource in China, with excellent soil fertility and production potential [1]. However, long-term monoculture planting leads to soil structure damage and a decrease in organic matter content, seriously affecting the sustainable use of arable land [2]. Crop rotation, as an important cultivation measure, can improve soil physical and chemical properties and enhance soil quality through the rotation of different crops [3]. In recent years, with the increasingly prominent issue of food security in China, protecting and improving the quality of black soil has become a national strategic task [4]. For a long time, corn monoculture has been the dominant planting mode in the black soil area. Although it ensures short-term high yield, it also leads to problems such as decreased soil organic matter, disrupted grain structure, and increased soil compaction [5]. The crop rotation system can not only break the cycle of pests and diseases through the cultivation of diversified crops, but also regulate the structure of soil microbial communities through the diversity input of root exudates and residues from different crops, thereby affecting the transformation and accumulation process of soil organic carbon [6]. At present, research on the impact of crop rotation on soil organic carbon mainly focuses on total changes, while there is relatively little research on its active components and their relationship with soil structure. Exploring the effects of different crop rotation patterns on soil organic carbon and its components has important theoretical and practical significance for the protection and efficient utilization of black soil.

In this study, we systematically analyzed the relationship between continuous cropping and crop rotation systems on soil organic carbon storage, active components, and soil aggregate stability through long-term positioning experiments, aiming to provide scientific basis and technical support for the sustainable development of agriculture in black soil areas.

## Materials and methods

### General situation

The experiment was conducted in the Soil and Fertilizer Experimental Park of Mudanjiang Branch of Heilongjiang Academy of Agricultural Sciences from 2020 to 2021. The experimental area is located at 44 ° 60 ′ N, 129 ° 58 ′ E, with a temperate continental monsoon climate, rain and heat in the same season, a frost free period of about 130 days, an average annual precipitation of 500–600 mm, an average annual temperature of 5.0 °C, and a soil type of dark brown soil. The basic characteristics of the soil in the experimental area are organic matter of 58.35%, pH 7.46, total nitrogen 1.835%, total phosphorus 0.438%, total potassium 0.406%, organic carbon 33.84%, alkaline hydrolyzed nitrogen 183.7 mg/kg, available potassium 164.0 mg/kg, bulk density 1.36 g/cm^3^.

### Tested materials

The main crop varieties planted in the black soil area were selected for the planting experiment. The corn variety was selected as “ Lvdan 4”, with a sowing rate of 25 kg/hm^2^ and a sowing density of 55–60000 plants/hm^2^. Urea (N 46%) was used as nitrogen fertilizer, diammonium phosphate (N 18%, P_2_O_5_ 46%) as phosphorus fertilizer, potassium chloride (K_2_O 60%) as potassium fertilizer, and potato variety was selected as “Youjin”. The integrated cultivation mode of water and fertilizer under film drip irrigation was adopted, with ridge spacing of 0.8 m, plant spacing of 0.25 m, drip head spacing of 0.2 m, flow rate of 1–2 L/h. Urea (N 46%) was used as nitrogen fertilizer, superphosphate (P_2_O_5_ 45%) as phosphorus fertilizer, and potassium chloride (K_2_O 60%) as potassium fertilizer. The soybean variety selected is “Mudou 14”, with a ridge spacing of 0.65 m and a plant spacing of 0.25 m. A 50% special fertilizer (N-P_2_O_5_-K_2_O=13-26-11) is used for fertilization. Rain fed planting is adopted during the growth period of corn and soybean seasons, without irrigation. All three crops start sowing in mid-May each year and are harvested in early October. Soil samples are collected using multi-point sampling method, with 5 sampling points set up in each community to collect soil layers of 0–50 cm. A soil sample is taken every 10 cm for analyzing soil physical and chemical properties at different depths.

### Experimental design

The experiment set up four treatments: corn continuous cropping (CCC), soybean continuous cropping (SSS), corn-soybean (CS) and corn-soybean-potato (CSP) rotation. Since the establishment of the experimental area in 2006, continuous planting has been carried out for 18 years. The corn soybean potato rotation has completed six rounds. The experiment adopted a random block design, with each treatment repeated three times. A total of nine plots were set up, each with an area of 156 m^2^ (12 ridges, ridge width of 0.65 m, ridge length of 20 m). The experimental treatments were uniformly leveled in autumn every year. After crop harvest, crop straw was crushed to a length of 5–10 cm using a crop cutter, and plowed and buried to a depth of 30–35 cm in the soil layer. During the corn season, nitrogen phosphorus potassium compound fertilizer was applied with a nitrogen phosphorus potassium ratio (N-P_2_O_5_-K_2_O) of 90-60-130 kg/hm^2^. During the potato season, the integrated cultivation technique of drip irrigation under plastic film was used, with the same amount of nitrogen, phosphorus, and potassium as in the corn season. During the soybean season, a specialized compound fertilizer with a nitrogen content of 50% was applied. To maintain consistency in experimental conditions, field management measures were uniformly implemented for each treatment. Soil samples were taken at depths of 0–50 cm affected by tillage, with samples taken every 10 cm. Five sampling points were selected for each treatment, and after sampling, soil samples at the same depth were mixed together as the corresponding soil layer for that treatment.

### Soil sampling and analysis

The soil samples were collected after the crop harvest on October 10, 2023. Five sampling points were set up in each community, and the soil samples were collected in layers of 0–10 cm, 10–20 cm, 20–30 cm, 30–40 cm, and 40–50 cm using the ring knife method. The five soil samples from the same treatment and layer were mixed and used as the test samples for that treatment and layer. The collected soil samples were divided into two parts: one part was directly used for soil bulk density and moisture content determination, and the other part was brought back to the laboratory for air drying and passed through a 2 mm sieve for soil chemical properties and aggregate analysis. The soil bulk density is determined by the ring knife method, the soil moisture content is determined by the drying method, the organic carbon is determined by the potassium dichromate external heating method, the total nitrogen is determined by the Kjeldahl nitrogen determination method, and the soil aggregates are analyzed by the wet sieve method using a water stable particle analyzer. The soil sample is classified through a series of different aperture sieves (2 mm 0.25 mm 0.053 mm), and the content of each particle size aggregate is determined. The proportion of water stable aggregates is calculated, and the changes in soil physical and chemical properties and organic carbon components under different planting modes are analyzed by measuring various indicator data.

### Measurement indicators and methods

The total porosity of soil is calculated using the formula: total porosity (%)=(1-soil bulk density/soil specific gravity) × 100. The natural soil specific gravity is 2.65 mg/m³, and the three-phase ratio of soil is obtained through calculation: solid phase: liquid phase: gas phase=(1-soil total porosity): (soil mass moisture content × bulk density): [soil total porosity – (soil mass moisture content × bulk density)]. The generalized soil structure index is calculated using the formula GSSI=[(Xg-25) XyXq] × 0.4769, where Xg is the solid phase volume percentage (>25%), Xy is the liquid phase volume percentage (>0), and Xq is the gas phase volume percentage (>0). The content of soil water stable aggregates is determined by wet sieving method, and the proportion of water stable aggregates with a content of >0.25 mm (WR0.25) is calculated as follows: WR0.25 (%)=Mr > 0.25/MT × 100, where Mr > 0.25 is the mass of aggregates with a particle size greater than 0.25 mm, MT is the total mass of aggregates, soil organic carbon is determined by potassium dichromate volumetric method external heating method, soil total nitrogen is determined by Kjeldahl nitrogen determination method, soil bulk density is determined by ring knife method, soil moisture content is determined by drying method, and all data measurements are repeated three times.

### Data processing

Statistical analysis was conducted on the experimental data, and one-way ANOVA was used to test for significant differences between treatments (*P* < 0.05), to analyze the relationship between soil physical and chemical properties, planting patterns, and soil depth, study the correlation between soil organic carbon and its components and soil structure indicators, and explore the influence of planting patterns and soil depth on soil aggregate composition. The data analysis results are presented as mean ± standard deviation, and the differences between different treatments and the degree of correlation between soil properties are shown through bar charts and correlation heat maps.

## Results

### Soil physical and chemical properties and organic carbon content

**Fig 1:**
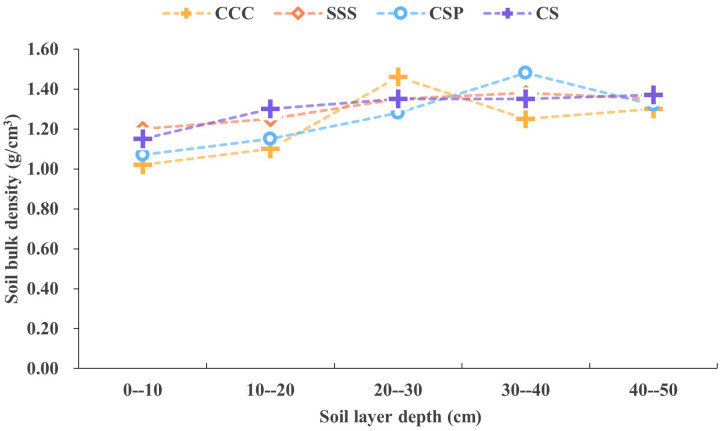
Changes in soil bulk density under different crop rotation treatments.

**Fig 2:**
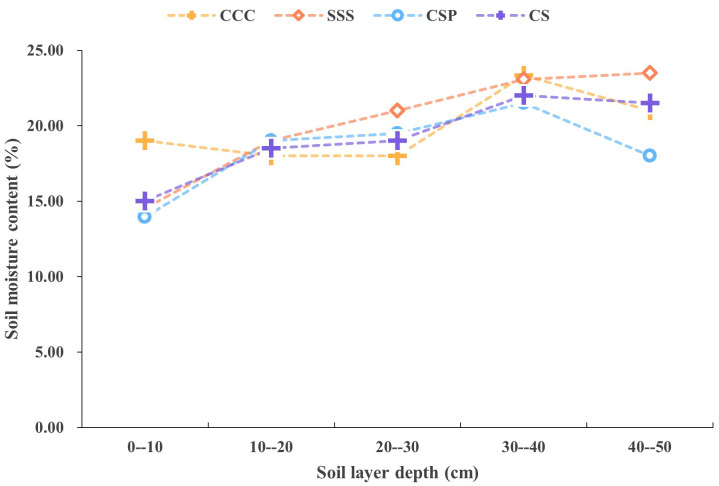
Changes in soil moisture content under different crop rotation treatments.

There are significant differences in soil bulk density and moisture content among different planting modes (Figs 1 and 2). From the distribution of soil bulk density, in the 0−10 cm plow layer, the soil bulk density of SSS treatment is 1.20 g/cm³, significantly higher than that of CSP treatment at 1.07 g/cm³ and CCC treatment at 1.02 g/cm³. As the soil layer deepens, the soil bulk density of CSP treatment reaches the maximum value of 1.48 g/cm³ in the 30−40 cm soil layer, while CCC treatment reaches the peak value of 1.46 g/cm³ in the 20−30 cm soil layer. The three treatments tend to stabilize in the 40−50 cm soil layer, and the bulk density remains between 1.30–1.37 g/cm³.

The characteristics of soil moisture content show that at the surface layer of 0–10 cm, the moisture content of CCC treatment reaches 19.02%, significantly higher than that of CSP treatment at 13.95% and SSS treatment at 14.53%. CSP treatment forms a significant water retaining layer in the 30–40 cm soil layer, with a moisture content of 21.51%, while CCC and SSS treatments at the same layer are 23.32% and 23.07%, respectively. Throughout the entire soil profile, CSP treatment exhibits a more uniform distribution of moisture, which is conducive to crop water absorption and utilization. The inter subject effect test showed that the effects of planting mode and soil depth on soil bulk density and moisture content reached a significant level (*P* < 0.01). These results indicate that different planting modes form their own unique soil physicochemical characteristics by affecting soil structure and moisture distribution. Among them, CSP rotation treatment showed significant advantages in improving soil structure and moisture regulation.

### Organic carbon active components

**Fig 3:**
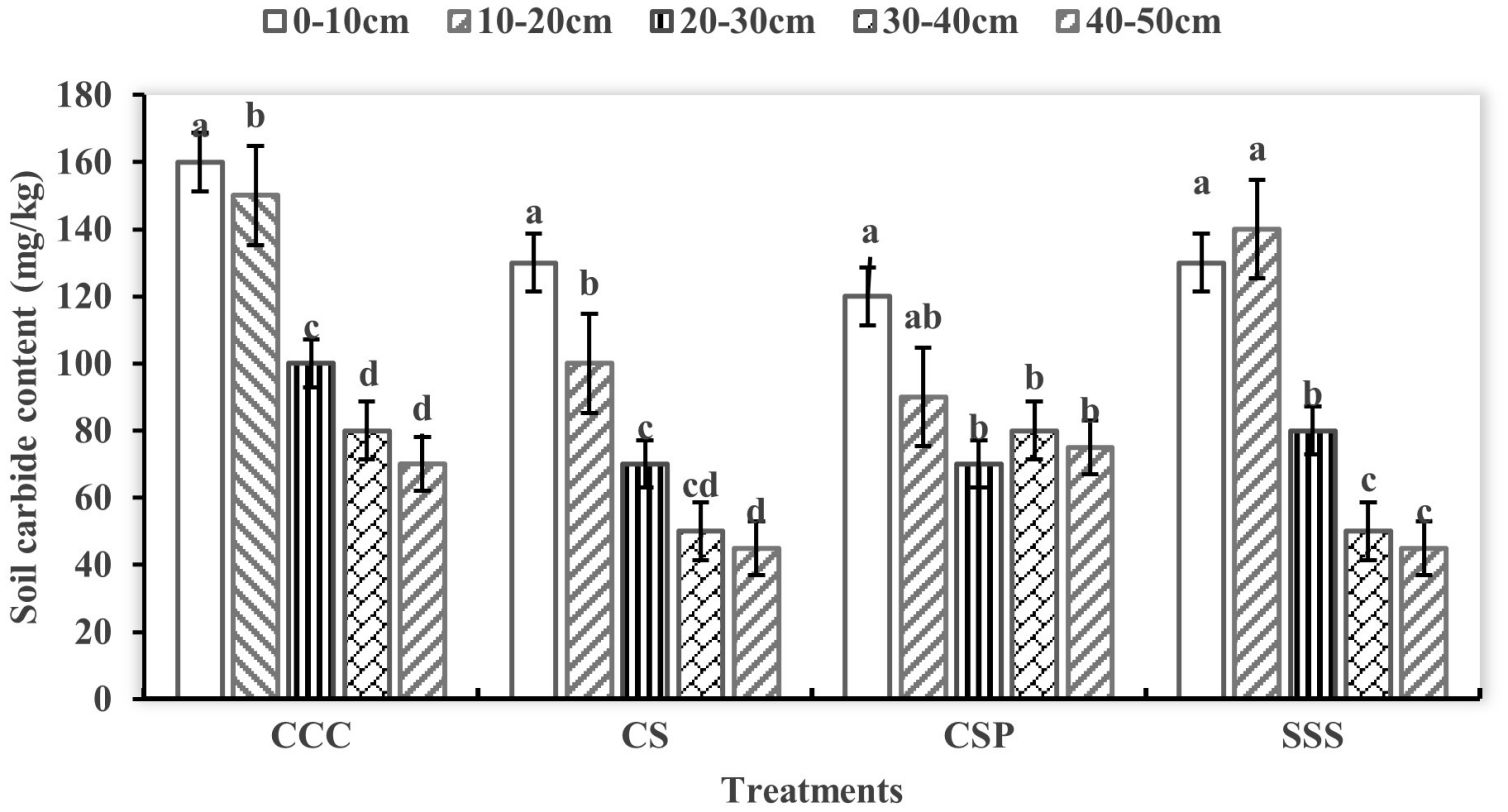
Changes in soil high active carbon content under different crop rotation treatments.

**Fig 4:**
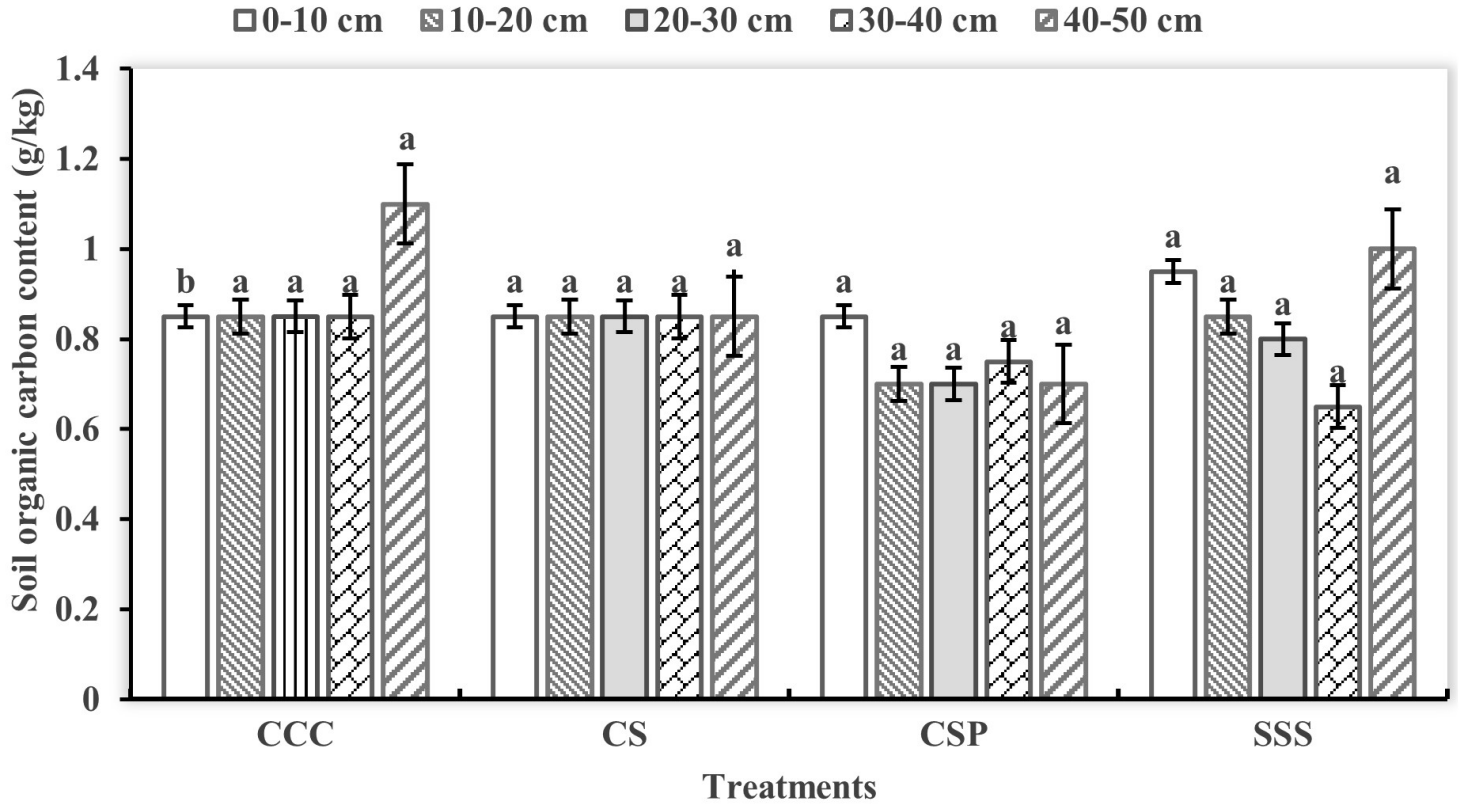
Changes in soil moderate active carbon content under different crop rotation treatments.

There are significant differences in soil active organic carbon components under different planting modes. Microbial organic carbon has the highest content in the 0–30 cm soil layer treated with CSP, maintaining between 0.63–0.68 g/kg and evenly distributed vertically (Fig 3). The CS treatment showed a decreasing trend with the deepening of the soil layer, from 0.63 g/kg in the surface layer to 0.45 g/kg in the 40–50 cm layer. The microbial organic carbon content in each soil layer was relatively low in the CCC and SSS treatments, but the Y treatment showed a more uniform vertical distribution. The difference in soil soluble organic carbon among the treatments was small, but at a depth of 40–50 cm, the content of CCC and SSS treatments was relatively high, reaching 1.2 and 1.1 g/kg, respectively (Fig 4). Relevant analysis shows that there is a significant positive correlation between microbial organic carbon content and soil aggregate stability (P < 0.05), indicating that crop rotation improves soil structure by increasing microbial carbon activity. This result reveals the positive effect of crop rotation on enhancing soil carbon activity and improving soil structure.

### Organic carbon storage and carbon stock index

The soil organic carbon storage under different planting modes shows differentiated characteristics in profile distribution (Table 1). The organic carbon storage in the 0–10 cm and 10–20 cm soil layers of CCC treatment reached 13.83 g/kg and 15.00 g/kg, respectively, significantly higher than other treatments. The organic carbon storage in the 20–30 cm soil layer of the CSP treatment was 14.84 g/kg, which was 12.5% higher than that of the continuous cropping treatment. The soil carbon pool index reflects the soil carbon accumulation and transformation ability. The average carbon pool index of the CSP treatment reached 99.60 in the 0–30 cm soil layer, indicating its strong carbon sequestration ability. The carbon pool index of the SSS treatment showed an increasing and then decreasing trend with the deepening of the soil layer, reaching the highest value of 93.79 at the 20–30 cm depth. The soil carbon storage is closely related to the distribution of soil aggregates. Soil layers with higher carbon storage indices often have more stable aggregate structures. Main effect analysis shows that planting patterns and soil depth have significant effects on soil carbon storage indices (*P* < 0.05), and there is an interaction between the two. Crop rotation systems optimize soil carbon storage structures by changing the distribution characteristics of soil carbon storage.

**Table 1:** Soil carbon pool index under different crop rotation treatments.

Soil layer depth (cm)	CCC	SSS	CSP
0-10	83.79b	81.47b	93.39a
10-20	88.79b	95.06ab	99.36a
20-30	93.00a	93.79a	99.60a
30-40	98.77a	97.40a	96.94a
40-50	95.90a	95.48a	96.46a

### Distribution of soil carbon nitrogen ratio

The soil carbon nitrogen ratio, as an important indicator reflecting the decomposition and transformation of soil organic matter and nutrient release, exhibits unique distribution patterns under different planting modes (Table 2). The soil carbon nitrogen ratio in the surface layer of 0–10 cm under CCC treatment is the highest, reaching 27.38, and gradually decreases with depth, reaching 18.50 at 40–50 cm. The variation range of carbon nitrogen ratio in each soil layer of SSS treatment is relatively small, maintaining between 15.50–16.80, showing a relatively stable vertical distribution characteristic. The carbon nitrogen ratio in the CSP treatment remains relatively balanced throughout the entire profile, with an average value of 17.34, and there is little fluctuation between each soil layer. Correlation analysis shows that the soil carbon nitrogen ratio is significantly negatively correlated with soil microbial activity (*P* < 0.05), and treatments with lower carbon nitrogen ratios often have higher microbial activity and organic matter decomposition rate. Through analysis of variance, it was found that the effect of planting mode on soil carbon nitrogen ratio reached a highly significant level (*P* < 0.01), and the effect of soil depth reached a significant level (*P* < 0.05). Crop rotation mode adjusted soil carbon nitrogen ratio, changed the characteristics of soil organic matter decomposition and transformation, and thus affected soil nutrient supply capacity.

**Table 2:** Soil carbon nitrogen ratio under different crop rotation treatments.

Soil layer depth (cm)	CCC	SSS	CSP
0-10	27.38a	16.80b	17.88b
10-20	24.50a	16.20b	17.50b
20-30	22.10a	15.90b	17.30b
30-40	19.80a	15.70c	17.10b
40-50	18.50a	15.50b	16.90ab

## Discussion

### Multidimensional impact mechanism of crop rotation mode on soil physicochemical properties

The long-term effects of different crop rotation patterns on soil physicochemical properties are manifested as multidimensional systematic changes [7]. The rotation of corn, soybean, and potato significantly improves soil structure, which is mainly due to the complementary effect of the root characteristics of three different crops. Corn has a developed whisker like root system that can be widely distributed in the surface soil [8], soybeans have a strong main root system that can penetrate deep into the lower soil [9], and potatoes have a physical loosening effect on the soil through their special tuber growth mode [10]. This complementary effect of root systems leads to a more rational soil pore structure, thereby reducing the bulk density of the 0–40 cm soil layer and improving the soil’s water storage and retention capacity. It is particularly noteworthy that the water retention layer formed at 30–40 cm may be related to the vertical distribution changes of soil organic matter caused by long-term crop rotation [11]. This area is located in the transition zone between the cultivated and non-cultivated layers, where the cumulative effect of root exudates from different crops in the rotation system is most obvious. This cumulative effect promotes the formation of soil aggregates, improves soil structure, and enhances soil water retention performance [12]. This is consistent with previous research on the improvement of soil aggregate structure through long-term crop rotation [13]. In addition, the soil three-phase ratio under the corn-soybean-potato rotation treatment is closer to the ideal value of 2:1:1, indicating that the crop rotation system can optimize the soil solid liquid gas three-phase structure, which is crucial for crop growth. A good soil three-phase structure is not only beneficial for crop root growth and nutrient absorption, but also provides a suitable living environment for soil microorganisms, promotes the increase of soil biological activity, and forms a virtuous cycle mechanism [14].

### Regulatory mechanism of crop rotation mode on soil carbon cycling

The effect of crop rotation mode on soil organic carbon and its active components reflects the differential regulation of soil carbon cycling by different crops [15]. The corn-soybean-potato rotation treatment significantly increased soil active carbon content, especially in the 20–30 cm soil layer, where microbial carbon and soluble organic carbon content increased by 31.8% compared to continuous cropping treatment. This increasing phenomenon can be explained from three aspects: firstly, leguminous crops (soybeans) provide abundant nitrogen sources through rhizobium nitrogen fixation, improving soil carbon nitrogen ratio and promoting microbial activity [16]; secondly, different crops have different root exudates, forming diverse carbon source supply, which is conducive to the formation of diverse microbial communities [17]; thirdly, the diversity of crop residues in crop rotation systems increases the complexity of organic matter decomposition pathways and prolongs the retention time of carbon in the soil [18]. This result is consistent with previous research findings on crop rotation improving soil microbial diversity and activity [19]. It is worth noting that although the total organic carbon content in the surface soil of corn continuous cropping is high, the proportion of active carbon components is low, indicating that its organic matter stability is strong but its activity is weak. This may be related to the high carbon nitrogen ratio and slow decomposition of corn straw, resulting in organic carbon accumulation but low conversion rate, which is not conducive to microbial utilization [20]. In contrast, the soil carbon pool index of soybean continuous cropping treatment changes less with depth, showing a relatively stable vertical distribution characteristic, which is closely related to the depth characteristics of soybean root distribution and nitrogen fixation [21]. In-depth analysis shows that there is a significant positive correlation between soil active carbon components and microbial activity, which means that crop rotation increases microbial activity by increasing soil active carbon content, thereby promoting soil nutrient cycling and organic matter transformation [22]. This transformation process is of great significance for maintaining soil fertility and improving agricultural productivity.

### Long-term shaping effect of crop rotation on soil carbon pool structure

The impact of crop rotation on soil organic carbon storage is not only reflected in the total amount, but also in the optimization of vertical distribution patterns [23]. The soil carbon pool index of the corn-soybean-potato rotation treatment reached 99.60 in the 0–30 cm soil layer, which is much higher than that of the single crop continuous cropping treatment. This indicates that crop rotation can significantly improve soil carbon sequestration capacity, especially in the 20–30 cm soil layer, where the organic carbon storage reaches 14.84 g/kg, an increase of 12.5% compared to continuous cropping treatment. This suggests that crop rotation is beneficial for deep soil carbon accumulation. The optimization of this vertical distribution pattern may be based on the following mechanisms: on the one hand, different crop root depths complement each other, forming a wider rhizosphere environment and expanding the range of organic matter input. On the other hand, crop rotation promotes soil microbial diversity, enhances the ability of organic matter decomposition and transformation, and makes the distribution of organic carbon more uniform in the profile [24]. In addition, crop rotation improves soil structure, reduces the risk of organic matter loss, and increases carbon sequestration efficiency [25]. There is a significant positive correlation between soil organic carbon storage and aggregate stability. Soil layers with higher carbon pool indices usually have more stable aggregate structures [26]. This correlation indicates that the optimization of soil carbon pool structure and the improvement of soil physical properties are mutually reinforcing processes, and crop rotation improves soil carbon fixation capacity and structural stability through this positive feedback mechanism. In addition, under crop rotation treatment, the soil carbon nitrogen ratio is maintained at a relatively stable level (average 17.34), which is within the suitable range for microbial decomposition of organic matter, and is conducive to nutrient release and organic matter transformation. This stable carbon nitrogen ratio is of great significance for maintaining soil microbial activity and promoting nutrient cycling. Overall, crop rotation not only affects the total amount of soil carbon pool, but more importantly optimizes the structure and quality of carbon pool, including the proportion of active components, vertical distribution pattern, and carbon nitrogen coupling relationship. This comprehensive optimization has long-term benefits for improving soil quality and promoting sustainable agricultural development.

### Comprehensive ecological benefits of crop rotation system in sustainable utilization of black soil

From the perspective of ecosystem services, crop rotation has multiple ecological benefits for black soil. Firstly, by improving soil structure and enhancing aggregate stability, crop rotation significantly enhances soil erosion resistance, which is of great significance in preventing black soil degradation [27]. Secondly, by optimizing soil carbon and nitrogen cycling, crop rotation improves soil nutrient utilization efficiency, reduces fertilizer dependence, and lowers agricultural non-point source pollution risks [28]. Thirdly, by increasing soil biodiversity, crop rotation enhances soil ecosystem stability and resilience, which is conducive to addressing climate change challenges [29]. In the long run, crop rotation, as an eco-friendly farming model, can maintain high crop yields and soil health, achieving a win-win situation between agricultural production and ecological protection, and contributing to the sustainable development of black soil. By providing a scientific pathway, it is recommended to promote diversified crop rotation patterns such as corn soybean potato in black soil areas, and combine straw returning and conservation tillage techniques to form a comprehensive soil management system, comprehensively improving the quality and productivity of black soil.

## Conclusion

Long term positioning experiments have shown that the rotation of corn-soybean-potato significantly improves soil physicochemical properties and organic carbon composition characteristics. By changing the soil structure and crop rotation treatment, the bulk density of the 0–40 cm soil layer was reduced, and a significant water retaining layer was formed at 30–40 cm. The soil three-phase ratio was closer to the ideal value of 2:1:1. Crop rotation increased soil active carbon content, with microbial carbon and soluble organic carbon content in the 20–30 cm soil layer increasing by 31.8% compared to continuous cropping treatment, and the soil carbon pool index reaching 99.60. The soil carbon nitrogen ratio maintained a relatively stable distribution in crop rotation treatment, with an average value of 17.34, indicating that crop rotation is beneficial for the decomposition and transformation of soil organic matter. Although corn continuous cropping has high organic carbon storage in the surface soil, the proportion of active components is low and the vertical distribution is uneven. The carbon stock index of soybean continuous cropping treatment changes little with depth, and the soil carbon nitrogen ratio remains stable. Crop rotation significantly enhances soil carbon sequestration capacity by regulating soil carbon pool structure and carbon active components. The research results provide scientific basis for soil fertilization and improvement of farmland quality in black soil areas.
